# High-Voltage Electrical Injury Causing a Full-Thickness Laryngotracheal Wall Defect: Reconstruction With an Anterolateral Thigh Free Flap and an Inverted Split-Thickness Skin Graft

**DOI:** 10.7759/cureus.116090

**Published:** 2026-09-11

**Authors:** Aparna Sinha, Shashank Chauhan, Nandini Singh Tanwar, Akshara Palreddy

**Affiliations:** 1 Plastic and Reconstructive Surgery, All India Institute of Medical Sciences, New Delhi, IND

**Keywords:** airway reconstruction, airway surgeries, anterolateral thigh flap, electrical burns, inverted skin graft, tracheal defect, tracheal wall loss

## Abstract

Full-thickness loss of the laryngotracheal wall is a rare and severe consequence of high-voltage electrical injury. It is anatomically and clinically distinct from the epithelialised tracheocutaneous fistula that follows tracheostomy decannulation, and it poses a far greater reconstructive problem because of progressive tissue necrosis, wound contamination, and exposure of the airway and major cervical vessels. We report the case of a 30-year-old man with approximately 30% total body surface area deep electrical burns involving the neck, abdomen, and all four limbs, who presented with an exposed airway and exposed major cervical vessels. After resuscitation and staged debridement, a full-thickness defect of the anterior and left anterolateral laryngotracheal wall was identified, extending from the cricoid to the fourth tracheal ring, with the glottis and vocal cords uninvolved and the membranous posterior wall intact. Reconstruction was performed on day 49 after injury with an inverted split-thickness skin graft as luminal lining, its raw surface applied to the deep fascia of a left anterolateral thigh (ALT) free flap anastomosed to the left transverse cervical vessels. An 18 Fr Foley catheter was left transnasally across the reconstructed segment as a through-and-through stent. No cartilage support was used. The postoperative course was uneventful, with complete flap survival, no recurrence of infection, and no fistula formation. Airway support was withdrawn in stages: extubation on the day of surgery, stent removal on postoperative day 14, and removal of the tracheostomy tube on postoperative day 21, after which the patient breathed through the reconstructed segment and his native upper airway alone. At six months, he had normal swallowing, near-normal speech, and 50 degrees of neck extension, with no stridor or exercise limitation; bronchoscopy, cross-sectional imaging, and formal voice assessment were not performed. In this single patient, an ALT free flap combined with an inverted split-thickness skin graft was a feasible option for a large laryngotracheal wall defect after severe electrical burn. Longer follow-up with objective assessment of airway patency and further cases are required before durability and reproducibility can be established.

## Introduction

Electrical burns cause extensive deep tissue destruction and carry substantial morbidity. Unlike thermal burns, electrical injury frequently involves deeper structures including muscle, cartilage, vascular tissue, and the airway [1,2]. When the cervical soft tissues are destroyed, the larynx, trachea, and great vessels of the neck may be exposed, and airway reconstruction becomes part of burn wound management.

The terminology used for cervical airway defects in the burn literature is inconsistent, and this affects how cases are identified and compared. A tracheocutaneous fistula is an epithelialised tract between the airway lumen and the skin, most often persisting after tracheostomy decannulation, in which the airway wall is largely intact, and the problem is failure of a tract to close. A tracheo-oesophageal fistula is an abnormal communication between the trachea and the oesophagus whose principal risk is aspiration. A full-thickness laryngotracheal wall defect is different from both: it is segmental loss of the airway wall, with the lumen open to the surface and no epithelialised tract, and the problem is absence of tissue. Only the last has a defect that can meaningfully be measured, which is why size is so rarely reported in the fistula literature.

The distinction matters most in electrical injury, where progressive coagulative necrosis destroys the airway wall together with the strap muscles, sternocleidomastoid, hyoid, and overlying skin -- the same locoregional tissue that would ordinarily be used for reconstruction. Strategies developed for post-tracheostomy fistula in an otherwise healthy neck do not transfer to a large, contaminated defect in a compromised donor field.

Reported approaches to cervical airway defects include conservative management, local tissue rearrangement, direct closure, and regional flaps such as the tunnelled supraclavicular artery island flap [3-8]. For true wall loss, published reconstructions have relied on prefabricated free flaps carrying costal cartilage, or on a pedicled flap used as a vascularised bed for cartilage grafts, generally in elective oncological or post-irradiation settings [9-13]. A separate body of work addressing narrowing rather than loss contributes the principle of relining the lumen with a split-thickness skin graft held by a stent [14,15]. Reports of large full-thickness laryngotracheal wall defects caused by high-voltage electrical burns remain scarce.

We report the reconstruction of a full-thickness laryngotracheal wall defect extending from the cricoid to the fourth tracheal ring following a high-voltage electrical burn, using an inverted split-thickness skin graft as luminal lining beneath an anterolateral thigh free flap, with a transnasal stent and without cartilage support. Neither component is itself new. In the search described further, we did not find this combination reported for an acute, contaminated airway defect caused by electrical injury.

This work was previously presented as a meeting abstract at the 6th Congress of the Asian Pacific Federation of Societies for Reconstructive Microsurgery (APFSRM 2024), held in conjunction with the 16th Biennial Conference of the Indian Society for Reconstructive Microsurgery, Delhi, India, 7-10 March 2024.

## Case presentation

A 30-year-old man sustained a high-voltage electrical contact injury, with approximately 30% total body surface area deep burns involving the neck, abdomen, both upper limbs, and both lower limbs. He was resuscitated at a district facility and passed through two further hospitals before referral to our unit. At the second of these, he required mechanical ventilation and a tracheostomy, and an escharotomy of the neck and hand was performed on day 1 after injury. His course there was complicated by sepsis, acute kidney injury, type 1 respiratory failure, hepatic dysfunction, and newly diagnosed hypertension. He had no pre-existing comorbidities. A wound swab on day 8 grew *Escherichia coli,* and culture-directed antimicrobial therapy was started. The tracheostomy was weaned on day 10, and a debridement under general anaesthesia was performed on day 11, after which he was referred to our institution.

He was admitted to our unit on day 11 after the injury. The neck wound was extensive, with exposed trachea and exposed major cervical vessels (Figure 1a). Negative-pressure wound therapy was continued over the neck wound between procedures. Management on admission was directed at haemodynamic stabilisation, control of infection, renal and hepatic support, and preparation of the wound bed.

**Figure 1:**
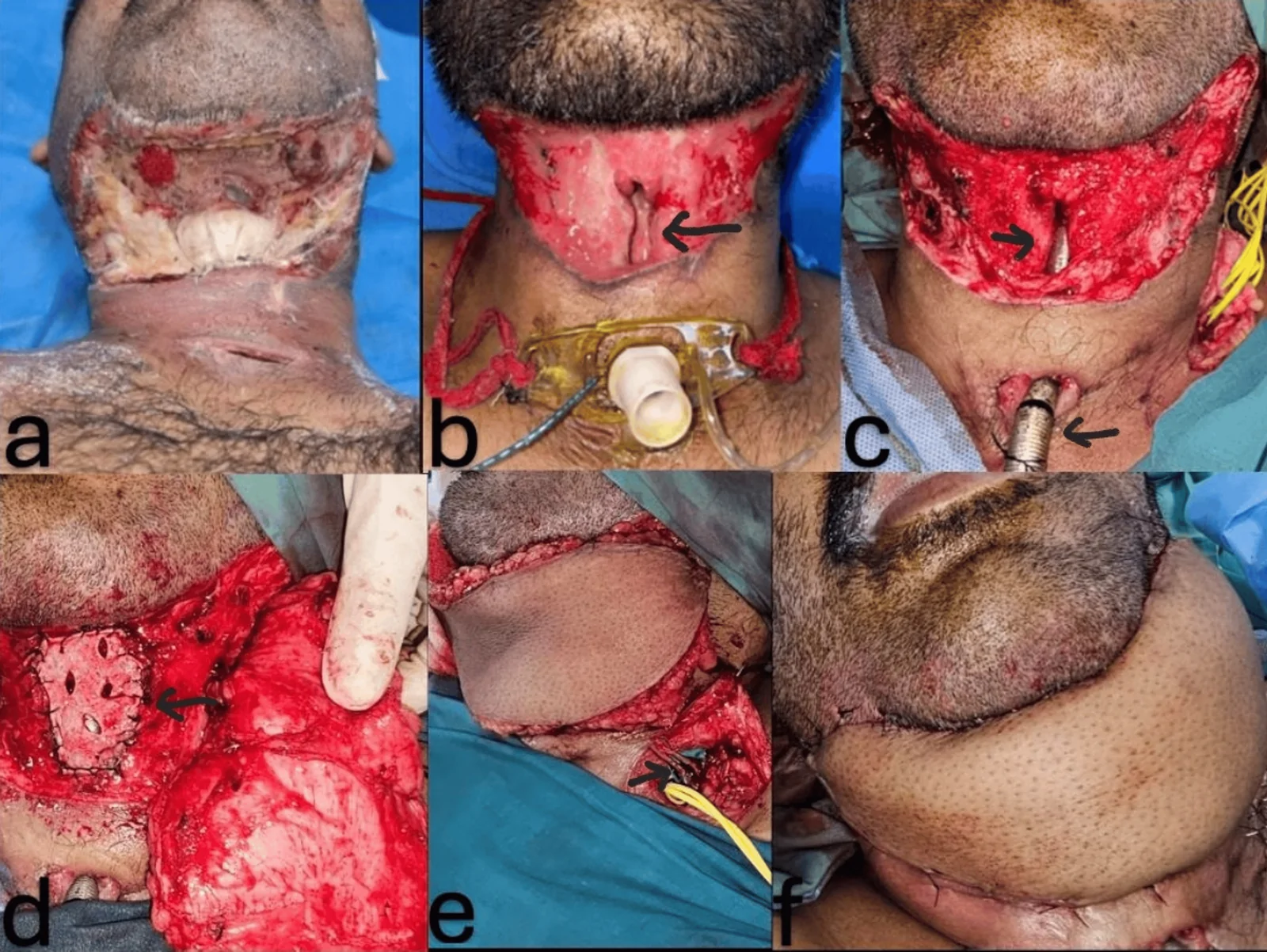
(a) Neck contact wound on the day of presentation, showing the exposed trachea and cervical vessels. (b) Wound after two debridement sessions (day 14 after injury), showing the 7 x 3 cm full-thickness anterior airway wall defect (arrow); note the exposed thyroid gland and the intact posterior membranous wall. (c) Upper arrow: flexometallic ET tube secured using railroad technique of catheter into tracheal defect to prevent tracheal closure; lower arrow: flexometallic tube through previous stoma for ventilation of patient. (d) Inverted split-thickness skin graft sutured over the tracheal defect. (e) Anterolateral thigh flap pedicle anastomosed to the left transverse cervical vessels (arrow). (f) Completed anterolateral thigh flap inset. ET, endotracheal.

A second debridement under general anaesthesia was performed on day 14 after injury. The necrotic bilateral strap muscles, both sternocleidomastoid muscles, the left submandibular gland, and the hyoid bone were excised. The left facial artery and vein were ligated, and the thyroid gland was exposed. Definitive reconstruction was undertaken on day 49 after injury, that is, 38 days after admission to our unit and 35 days after the second debridement. This interval was required for control of sepsis and recovery of renal and hepatic function. During this period, the wound was managed with regular bedside dressing and debridement.

A full-thickness airway wall defect measuring 7 cm × 3 cm was identified (Figure 1b). The defect involved the anterior and left anterolateral wall over approximately four rings, including the cricoid, extending from the cricoid to the fourth tracheal ring. The glottis and vocal cords were not involved. The membranous posterior wall was intact, there was no circumferential loss, and there was no communication with the oesophagus.

The ventilatory requirement had been weaned on day 10 after injury before referral to us; the tracheostomy tube was in situ, and the stoma was used for ventilation during the debridement procedures and during the definitive procedure using a flexometallic endotracheal (ET) tube, as shown in Figure 1c. Luminal support was therefore established by an ET tube introduced transnasally, railroaded over Ryle's tube passed through the open tracheal defect, and Ryle's tube was then removed (Figure 1c). This provided a through-and-through transnasal stent lying across the reconstructed tracheal segment. Ventilation during the procedure was maintained by the flexometallic tube placed through the tracheal stoma. The wound was then debrided to a healthy bleeding base.

A split-thickness skin graft (thickness 0.6 mm) was harvested from the right thigh and inverted, so that the epidermal surface faced the airway lumen and the raw undersurface faced outward. The graft was sutured circumferentially to the margins of the defect with interrupted sutures (Figure 1d). The graft was not dependent on the underlying bed for vascularity; it was intended to take from the vascularised deep fascia of the overlying free flap.

A left anterolateral thigh free flap was designed over the defect with the perforator marked centrally. The perforator and the descending branch of the lateral circumflex femoral artery were identified and dissected to their origin, and the flap was raised on the identified perforator. It measured 20 cm × 12 cm. The deep fascia of the flap was sutured to the edges of the defect over the inverted graft, so that the fascial surface lay directly against the raw undersurface of the graft. Microvascular anastomosis was performed to the left transverse cervical vessels with 9-0 nylon sutures (Figure 1e), and the flap was inset without tension (Figure 1f). At the same sitting, split-thickness skin grafts were harvested and applied to the granulating burn wounds of the abdomen, right thigh, and the left leg.

Intraluminal support after reconstruction was provided by removing the ET tube and placing an 18 Fr Foley catheter, which was retained as a transnasal stent and removed on postoperative day (POD) 14. The patient was extubated on POD 0, and the tracheostomy was removed on POD 21; from POD 21, the patient breathed through the reconstructed tracheal segment and his own upper airway, with the stent in situ and no artificial airway. The postoperative course was otherwise uneventful. The flap survived completely, and there was no recurrence of infection and no fistula formation.

At six months, swallowing was normal, assessed by clinical water swallow test and tolerance of a full oral diet without aspiration. Speech was near-normal. Neck extension was satisfactory, with 50 degrees of extension. Bronchoscopy and cross-sectional imaging of the airway were not performed, as the patient was asymptomatic and declined further invasive assessment.

## Discussion

Loss of the laryngotracheal wall after high-voltage electrical injury is uncommon but severe. Electrical current produces progressive tissue necrosis through vascular thrombosis and deep thermal damage, and in the neck, this can destroy the strap muscles, sternocleidomastoid, hyoid, and the overlying skin, leaving the airway and great vessels exposed [1,2].

We have deliberately described this lesion as a full-thickness laryngotracheal wall defect rather than a tracheocutaneous fistula. Our patient had no epithelialised tract; he had segmental loss of the anterior and left anterolateral wall extending from the cricoid to the fourth tracheal ring, with the lumen open to the surface, the glottis and vocal cords uninvolved, and the membranous posterior wall intact. The distinction is not semantic. A post-tracheostomy tracheocutaneous fistula is a failure of a tract to close in an otherwise intact airway, and local rearrangement or conservative measures usually suffice [4,5]; here the problem was the absence of tissue. The distinction also determines which literature applies, and it explains why defect size is so inconsistently reported in the fistula literature: a fistulous tract has no wall defect to measure.

Cricoid involvement raises the stakes further. The cricoid is the only complete ring in the airway and the sole rigid circumferential support of the subglottis, so partial loss removes the one structural element the trachea cannot compensate for. This is the region in which post-injury stenosis is most common and most difficult to treat, and it is why we regard the absence of cartilage in our reconstruction as a limitation rather than a simplification.

Initial management centred on haemodynamic stabilisation, infection control, airway maintenance, and debridement. Our patient required intensive care for sepsis, acute kidney injury, type 1 respiratory failure, and hepatic dysfunction, with an *Escherichia coli* wound infection, before reconstruction could be contemplated. Timing was decisive. Definitive reconstruction was deferred to day 49 after injury, 35 days after the last formal debridement, by which point the wound bed was clean and the systemic derangements had resolved. This is not merely pragmatic. In a burn microvascular series, overall flap failure was 13%, eight of 10 failures occurred between five and 21 days after trauma, all 10 fell between five days and six weeks, and none occurred during secondary reconstruction [16]. In acute electrical injury of the upper limb, flow-through anterolateral thigh flaps transferred at a mean of 5.8 days achieved 80% limb salvage but carried a higher failure risk than flaps for other burn aetiologies [17,18]. Reconstruction at day 49 lay outside the interval in which failure clusters. The corollary is that this strategy requires a patient who can be kept safe for several weeks with an open airway defect, negative-pressure dressings, and a tracheostomy in situ, which is itself a form of selection.

Locoregional options were considered first and were unavailable. The tunnelled supraclavicular artery island flap has an established role in tracheostomy defects [8], and pedicled myocutaneous flaps have been used successfully for acute electrical burn wounds of the neck and shoulder [19,20]. In our patient, the cervical and supraclavicular tissues lay within the burn field and had been debrided; both sternocleidomastoid and strap muscles had been excised, and burns over the chest and abdomen compromised the pectoralis major territory. Any locoregional flap would have brought tissue from within the zone of injury into a recently infected bed.

The published reconstructions of comparable full-thickness airway wall defects were absent from our original review and are worth setting out. Teng et al. implanted costal cartilage strips into a radial forearm free flap to replicate the anterior tracheal wall, using forearm skin as lining, for an 8 cm trough involving 40% of the anterior circumference in an irradiated neck [8]. Fujiwara et al. used a prefabricated, double-folded radial forearm flap containing costal cartilage to provide both lining and external cover for a 3.5 cm defect involving 60% of the circumference [10]. Nakahira et al. used a pectoralis major myocutaneous flap as a vascularised bed for free costal cartilage grafts to close a 55 × 30 mm cervico-mediastinal defect, the closest published size comparator [11]. Royer et al. closed a large tracheocutaneous fistula involving the entire anterior wall with a prefabricated composite radial forearm flap in an irradiated field, with no airway compromise at 60 months [12]. Zhi et al. reported the largest relevant series, with 62 patients undergoing laryngotracheal reconstruction with autogenous rib cartilage graft and stenting for complex laryngotracheal stenosis or anterior neck defect, of whom three with subglottic stenosis and an anterior neck defect were reconstructed in a single stage with rib cartilage interposition and a fasciocutaneous flap; 74.1% were decannulated [13].

The anterolateral thigh flap was selected for four specific reasons rather than size alone. Its long pedicle allowed anastomosis to the left transverse cervical vessels, well outside the zone of injury. It provided a large, thin, pliable paddle able to resurface a defect of this dimension in one stage. Its deep fascia offered a vascularised surface that could be applied directly against a skin graft, which is central to the technique described here. Donor-site morbidity was limited, and the thigh lay outside the principal burn areas of the neck and trunk. The anterolateral thigh flap has also proved reliable in the burned neck in other contexts, accounting for four of nine free flaps in a series of post-burn cervical contracture releases [21].

The decision to use no cartilage requires a fuller justification than we offered in the submitted version. Every published reconstruction of a comparable defect used cartilage for structural support, and the report by Zhi et al. shows that rib cartilage and a fasciocutaneous flap can be combined in a single stage for a cricoid-level defect with an anterior neck component [9-13]. Our reasoning was therefore not that a one-stage cartilage reconstruction is impossible, but that the recipient site would not tolerate it. Those series were performed in stable airways scarred by intubation, radiotherapy, or oncological resection. Our patient was reconstructed six weeks after a 30% total body surface area burn, in a bed that had grown *Escherichia coli*, in a neck from which the strap muscles, sternocleidomastoid, submandibular gland, and hyoid had been excised. A non-vascularised cartilage graft placed in such a bed carries a substantial risk of infection, resorption, and extrusion, and its loss would have exposed the airway and the cervical vessels a second time. We accepted the absence of a rigid framework in exchange for the lowest possible burden of avascular implanted tissue, and relied on a transnasal stent during healing and on the bulk and vascularity of the flap thereafter. This is a defensible trade-off in an infected field, not a general argument against cartilage, and in a clean bed we would not make the same choice.

The distinguishing feature of the reconstruction was the inverted split-thickness skin graft placed beneath the flap. In conventional grafting, the raw surface is laid against the recipient bed, from which the graft revascularises. Here, the vascular supply lay above rather than below, so the graft was inverted: its raw undersurface was applied to the deep fascia of the overlying flap, from which it took, while the epidermal surface faced the airway lumen. A relatively thick graft of 0.6 mm was chosen so that the lining would carry sufficient dermis to resist contraction and inward collapse during healing. The principle of lining an airway with skin is not new, and our claim of novelty requires qualification. Split-thickness grafting of the lumen over a stent is established in laryngotracheal stenosis: it is the basis of endoscopic laryngotracheoplasty, in which a graft relines the subglottis and is held by a silastic stent [15], and it has salvaged complex paediatric laryngotracheal stenosis after failed reconstruction [14]. Because our defect was itself laryngotracheal, that literature is directly analogous rather than merely adjacent. Skin has also served as a tracheal lining within prefabricated free flaps [9,10]. What we did not find described is this combination: an inverted split-thickness graft as a lining beneath a free anterolateral thigh flap, without cartilage, for an acute contaminated laryngotracheal wall defect caused by a high-voltage electrical burn.

One observation deserves more weight than we gave it in the submitted version. Airway support was withdrawn in stages, and the patient tolerated each step. He was extubated on the day of surgery, the transnasal stent was removed on POD 14, and the tracheostomy tube was removed on POD 21, after which he breathed through the reconstructed segment and his native upper airway with no artificial airway and no stent. Sustained spontaneous ventilation across a reconstructed subglottic and upper tracheal segment, maintained for six months without stridor, exercise limitation, or need for reintubation, is functional evidence that the lumen remained patent and did not collapse in the absence of cartilage. It is not equivalent to bronchoscopy, and we do not present it as such, but it is more informative than the absence of symptoms alone, and it is the principal reason we consider the technique worth reporting.

Functional outcome deserves separate comment because the defect involved the larynx. The glottis and vocal cords were spared, which is the most likely explanation for the recovery of near-normal speech: the reconstruction restored the subglottic and upper tracheal wall without disturbing the phonatory apparatus. Swallowing was normal on clinical water swallow testing with tolerance of a full oral diet and no aspiration, consistent with an intact glottis and no communication with the oesophagus. Neck extension measured 50 degrees. Voice was assessed clinically rather than by laryngoscopy or formal voice evaluation, which, in a reconstruction involving the cricoid, is a real gap.

The limitations of a skin-lined airway are real and should be stated plainly. A skin graft restores no respiratory epithelium and provides no mucociliary clearance, so crusting, retained secretions, and impaired mucus handling remain concerns over the long term. Secondary contracture of a graft in a tubular structure could contribute to stenosis, and graft loss over a contaminated bed would have risked re-exposure of the airway. None of these occurred during six months of clinical follow-up, but neither were they objectively excluded: bronchoscopy and cross-sectional imaging were not performed, and clinical assessment is not a substitute for direct visualisation of the reconstructed lumen. Burn patients remain at risk of recurrent infection, airway compromise, and late fistula formation, and surveillance matters [7].

The relevant literature is summarised in Table 1. The search was repeated for this revision after the terminology of the lesion was reconsidered. We searched PubMed, Scopus, and Google Scholar without date restriction, combining terms for the lesion, the reconstruction, and the aetiology, and screened the reference lists of retrieved articles. Restricting the original search to the word fistula had excluded the papers that are in fact the closest comparators, since reconstructions of true airway wall loss are reported under defect, fenestration, and laryngotracheal reconstruction. Table 1 is now organised by what was reconstructed rather than by what the lesion was called, and defect size is compared only within the category where it is meaningful. The journal's reference limit for case reports required us to omit several peripheral citations in favour of the directly comparable defects, and our claim of novelty should be read as a statement about what the search retrieved rather than an assertion that the technique has never been used.

**Table 1:** Reported reconstructions of airway wall defects, skin-lined airway repairs, and airway or neck defects in burn patients Defect size is reported only for lesions involving true loss of airway wall; for epithelialised fistulous tracts it is not an applicable measurement. TBSA, total body surface area.

S. No.	Study (year)	Lesion/defect	Population and aetiology	Reconstruction	Size and outcome
A. Full-thickness airway wall defect reconstructed with a flap (directly comparable defects)					
1	Teng et al., 2005 [8]	Anterior tracheal wall defect	Post-oncological resection; irradiated cervical skin precluded conventional tracheoplasty	Prefabricated composite radial forearm free flap with implanted costal cartilage strips; forearm skin used as tracheal lining; deltopectoral flap for skin cover; soft stent for 3 weeks with tracheostomy tube beneath; two-stage	8 cm trough involving 40% of the anterior tracheal circumference
2	Fujiwara et al., 2009 [10]	Anterior tracheal wall defect	Tracheal resection with total thyroidectomy for papillary carcinoma	Prefabricated, double-folded radial forearm free flap containing costal cartilage, providing both internal lining and external resurfacing; two-stage	3.5 cm long, 60% of the circumference
3	Nakahira et al., 2006 [11]	Cervico-mediastinal tracheal defect	72-year-old man; thyroid carcinoma with tracheal invasion and mediastinal nodal metastasis	Pectoralis major myocutaneous flap as a vascularised bed for two free costal cartilage grafts, rotated onto the trachea at a second stage	55 × 30 mm; closest published size comparator to the present case
4	Royer et al., 2015 [12]	Large tracheocutaneous fistula with tracheal wall loss and adjacent stenosis	66-year-old man; prior surgery and radiotherapy to the lower neck	Prefabricated composite radial forearm free flap: buccal mucosa lining, conchal cartilage framework, external skin; staged	Entire anterior wall and part of both sidewalls; no airway compromise or new dysphonia at 60 months
5	Zhi et al., 2014 [13]	Complex laryngotracheal stenosis and/or anterior neck defect	62 patients, including cricoid-level disease	Rib cartilage graft interposition with silicone stent (10 days to 12 months); 3 patients with subglottic stenosis plus anterior neck defect had single-stage rib cartilage graft with fasciocutaneous flap	46 of 62 (74.1%) successfully decannulated
6	Present case	Full-thickness anterior and left anterolateral laryngotracheal wall defect	One adult; high-voltage electrical burn, approximately 30% TBSA, infected	Inverted split-thickness skin graft as luminal lining, anterolateral thigh free flap cover, transnasal Foley stent; no cartilage support; single stage on day 49	Cricoid to fourth tracheal ring; extubated on day 0, stent removed on day 14, tracheostomy removed on day 21; no fistula or infection recurrence at 6 months
B. Skin graft used as luminal lining of the airway (precedent for the lining principle)					
7	Bowe et al., 2018 [14]	Complex laryngotracheal stenosis	Two children, after failed previous reconstruction	Split-thickness skin grafting with stenting; stent material varied with the clinical scenario	Decannulation or speech production achieved
8	Kavookjian and Hillel, 2023 [15]	Idiopathic subglottic stenosis	Adults	Endoscopic laryngotracheoplasty (Maddern procedure): endoscopic scar excision with split-thickness skin graft relining the lumen, held by a silastic stent	Established endoscopic technique; excludes dense posterior scar, glottic involvement, and long-segment stenosis
C. Airway, neck, and fistula defects in burn patients					
9	Seidl et al., 2008 [6]	Tracheal rupture with tracheo-oesophageal fistula	Two paediatric burn patients among 1693 ventilated; iatrogenic (cuff overinflation, oversized tube)	Collar approach with direct closure of the fistula and end-to-end tracheal anastomosis	Case 1: 5 cm posterior wall tear. Case 2: 7 cm posterior wall defect with 1 cm oesophageal fistula
10	Eipe et al., 2005 [3]	Tracheo-oesophago-cutaneous fistula	15-year-old boy; electrical burn to the neck	Airway control only: tracheostomy through the traumatic opening, intercostal drain, then formal low tracheostomy below fistula level	Size not reported; no reconstruction described
11	Mallik et al., 2024 [16]	Acute neck defect with exposed thyroid cartilage and carotid sheath	Acute electrical burn of the neck	One-stage pectoralis major myocutaneous flap reconstruction	13 × 10 cm; neck extension 100 degrees measured by goniometer at follow-up
12	Meng et al., 2020 [20]	Deep neck, shoulder, and back electrical burn wounds	Two adults, high-voltage injury	Lower trapezius myocutaneous flap	Up to 30 ×​​​​​​​ 12 cm; neck function and contour satisfactory
13	Ferland-Caron et al., 2023 [21]	Post-burn neck contracture	Nine burn patients, 20-70% TBSA	Free tissue transfer: four anterolateral thigh, four radial forearm, one ulnar forearm	All flaps successful; 5 of 9 required at least one secondary defatting procedure
14	Huber et al., 2017 ​​​​​​​[4]; Camargo et al., 2020​​​​​​​ [5]	Epithelialised post-tracheostomy tracheocutaneous fistula	Burn and non-burn patients after tracheostomy decannulation	Conservative wound care with grafting; multilayered local tissue rearrangement; hinged skin flaps	Not applicable: a fistulous tract has no wall defect to measure
D. Timing of free tissue transfer after electrical injury					
15	Baumeister et al., 2005 [17]	Free flaps in burn and electrical burn injury	Series of microvascular reconstructions	Predominantly cutaneous flaps; timing analysed against outcome	Overall flap failure 13%; 8 of 10 failures at 5-21 days, all 10 between 5 days and 6 weeks; no failure during secondary reconstruction
16	Hsiao et al., 2013 [18]	Acute electrical burn of the upper limb	Five men, high-voltage injury with segmental arterial defects	Flow-through anterolateral thigh free flap	Mean 5.8 days after injury; 80% limb salvage; higher failure risk than other burn aetiologies

Finally, this case illustrates the value of a multidisciplinary approach. Management required plastic and reconstructive surgeons, burn specialists, anaesthetists, and intensive care physicians working together on airway control, sepsis management, staged debridement, and microvascular reconstruction over a seven-week period before definitive coverage was possible.

Limitations

This is a single case with six months of follow-up. Airway patency was assessed clinically and functionally, not by bronchoscopy or imaging, and voice was assessed clinically rather than by laryngoscopy or formal evaluation, which is a particular limitation given cricoid involvement. The long-term behaviour of a skin-lined laryngotracheal segment without cartilage support, including contracture, subglottic stenosis, and secretion handling, remains unknown. The technique should be regarded as feasible in this patient rather than established.

## Conclusions

High-voltage electrical burns can produce full-thickness loss of the laryngotracheal wall with extensive soft tissue destruction and airway exposure. This lesion is distinct from post-tracheostomy tracheocutaneous fistula and should be described as such, since the reconstructive requirements differ: the problem is absence of tissue in a contaminated field, not failure of a tract to close. Management requires prompt stabilisation, infection control, staged debridement, and definitive coverage deferred until the wound bed is clean and the patient systemically recovered.

In this patient, an inverted split-thickness skin graft as luminal lining beneath an anterolateral thigh free flap, supported by a transnasal stent and without cartilage, allowed staged withdrawal of airway support with extubation on the day of surgery, stent removal on POD 14, and decannulation on POD 21, followed by unassisted breathing through the reconstructed segment at six months. The decision to omit cartilage was driven by the infected recipient bed and should not be generalised: in a clean field, the published reconstructions of comparable defects all incorporate structural support, and we would do the same.

Neither component of this technique is new, and we claim only that we did not find the combination reported for an acute contaminated laryngotracheal defect after electrical injury. On the evidence of a single case followed clinically for six months, without bronchoscopy, imaging, or formal voice assessment, this approach should be regarded as feasible rather than established. Further cases and longer follow-up with objective assessment of airway patency and voice are needed before its durability and reproducibility can be judged.
